# Decoupling the United States Medical Licensing Examinations (USMLEs) From the Medical Curriculum to Promote Student Well-Being and Professional Identity Development

**DOI:** 10.7759/cureus.83335

**Published:** 2025-05-02

**Authors:** Leon Liu, Nisha Chachad, Arash Tadjalli, Vijay Rajput

**Affiliations:** 1 Medical Education, Nova Southeastern University Dr. Kiran C. Patel College of Allopathic Medicine, Davie, USA; 2 Medical Education, Nova Southeastern University Dr. Kiran C. Patel College of Allopathic Medicine, Fort Lauderdale, USA

**Keywords:** curricular reform, professional identity formation, student well-being, undergraduate medical education, united states medical licensing examination

## Abstract

The United States Medical Licensing Examination (USMLE) program administers a series of longitudinal assessments across medical education to ensure that future physicians meet baseline competency for independent clinical practice. Through the current system of USMLE integration within medical school education, passing the Step 1 and 2 Clinical Knowledge (CK) exams is required for both application to residency programs and graduation from undergraduate medical education (UME). While the original intentions of such standardized exams were to establish benchmarks for medical knowledge and clinical competence, the current high-stakes system has diverged to the point that USMLE performance is now the single most defining factor utilized in residency consideration. Over-prioritization of USMLE exams by curriculum developers and program directors (PDs) alike has heightened stress levels and intensified reliance on parallel curricula, where the use of third-party resources far outweighs genuine engagement in formal medical school curriculum and education due to perceived inadequacy of classroom didactics in preparing students for board exams. For this reason, we have proposed the separation of USMLE from the medical school curriculum and the transition toward a new system where both Step 1 and 2 CK are postponed until after the residency match. In doing so, we aim to promote enhanced well-being of medical students and increased emphasis on holistic learning based on curiosity and intrinsic motivation for the practice of medicine. There may be unintended consequences to this proposition, particularly with regard to internal assessment of minimum standards for medical school graduation and changes in the residency application process. However, we hold that our proposal, combined with a reevaluation of criteria used in the residency selection process, would support well-roundedness among students and the full realization of their potential as the next generation of physicians.

## Editorial

Introduction

Professional licensing exams are the cornerstone of any profession. Medical licensing exams and specialty board exams are an established aspect of medical education at all levels. The United States Medical Licensing Examination (USMLE) is a three-step examination program for medical licensure and represents the only route of licensure in the United States for graduates of accredited medical schools and international medical schools offering Doctor of Medicine (MD) degrees [1].

In the early 1990s, a joint licensing exam system was developed and implemented by the National Board of Medical Examiners (NBME) to replace the previously dualized system involving the Federation Licensing Examination (FLEX) [1]. The original intention of the USMLE was to serve as a minimum competency assessment for the medical knowledge and skills necessary for clinical practice, providing an objective examination of physician competence to enter independent patient care [2]. As such, the USMLE has remained fluid over its decades of usage, adapting its design and content in response to evolving medical practice and demand for educational change [1].

Over time, the utilization of USMLE exams at the undergraduate medical education (UME) level has increased to the point that passing USMLE Step 1 and 2 Clinical Knowledge (CK) exams is required for progression throughout and graduation from medical school. USMLE performance is currently cited as one of the single most important criteria used by residency program directors (PDs) in considering applicants for interview and acceptance. This circumstance is in stark contrast to the 1970s, when NBME and FLEX examination performances were ranked in the lower half of factors used for ranking residency applicants [3]. Therefore, such standardized exams have become more of a screening tool than a meaningful method of educationally appraising contemporary medical students.

Medical educators have long argued that standardized exams should not be the sole basis for evaluating student competence, but rather one of the many assessment tools [4]. However, the growing importance of the USMLE in the residency matching process may be attributed to the perceived lack of objectivity of other metrics by PDs. For instance, the Medical Student Performance Evaluation (MSPE) and Letters of Recommendation (LORs) may be seen as subjective, and clerkship grades are often thought to be artificially inflated rather than accurate reflections of student clinical performance [5]. The phenomenon of overreliance on USMLE raises the question of whether it may be time to disconnect these exams from medical school curricula and graduation requirements entirely and instead utilize them as a benchmark for student competence. In this perspective paper, we propose that both Step 1 and 2 exams be deferred until after medical school graduation and the residency match process, hence excluding them as criteria for residency selection.

Impact of integrating MD graduation requirements and licensing exams 

Given the extreme emphasis on USMLE performance by medical curriculum developers and residency directors alike, the unintended growing impact of the “exam mania” on student stress and burnout is considerable and has altered the learning environment. This is reinforced by a “hidden curriculum” surrounding the USMLE exams; students learn from peers and faculty that the USMLE is the single most defining moment in their professional education and career choice [6]. The resulting high-stakes environment ties student extrinsic motivation, engagement, and perceived competence to exam performance [6], leading to anxiety, depression, and social deprivation while diminishing personal and professional gratification [7]. Elevations in stress levels have progressed to the point that many students seek mental health care or resort to using illicit prescription stimulants to cope with elevated stress levels [8]. Despite the partial relief of stress granted from the recent transition of the Step 1 exam to a pass/fail scoring system in 2020 [9], we remain in an era of unprecedented competitiveness among medical students to match into desired career residency programs. This phenomenon, known as “Step mania” [3] to describe the wildly cutthroat culture surrounding standardized examination, is more prevalent and toxic than ever.

Preparation for the USMLE has effectively become the “de facto” medical school curriculum, causing students to devote time to rote memorization of information they will discard after the exam [3]. This method results in reduced curiosity, critical thinking, understanding of disease mechanisms, and willingness to consider the biopsychosocial context of illness [10]. Prioritization of short-term learning strategies over long-term ones may lead to a decreased desire to fill gaps in knowledge [9], as well as a sense of complacency with not knowing medical science material that may consequently hinder critical reasoning skills. In response, medical schools have adopted a complementary small group curriculum that encourages students to practice critical reasoning and problem-solving skills while applying medical knowledge to navigate patient cases. Despite this attempt at mitigation, the enormous amount of medical information and the rapid-fire testing style of standardized exams remain in favor of achieving the right answer over the process of clinical reasoning. This need for instant gratification stems from students’ adoption of a perfectionist mindset and anxiety about underperforming [11], created by overemphasis on USMLE scores.

The emphasis on performing well on USMLE exams has led to a parallel curriculum that disengages students from formal medical education. Parallel curricula and the resulting reliance on commercial products have become a staple in medical schools. The use of resources such as First Aid, UWorld, Pathoma, Sketchy, and Anki that offer buzzword high-yield reviews to maximize exam scores is nearly ubiquitous among contemporary medical students [12,13]. In a 2022 survey conducted by the Association of American Medical Colleges (AAMC), 70% of second-year medical students used non-institution-created online resources daily or weekly for education, with less than 50% regularly attending pre-clerkship classes if not required to attend [13]. According to the 2022 AAMC Summary Report, 28.7% of students reported “almost never” and “occasionally” attending virtual clerkship courses, compared to 26.6% in 2021 and 25.0% in 2020 [14]. Therefore, disengagement with formal curriculum is an evident and growing issue.

The drive to engage in parallel curricula, particularly during pre-clerkship education, originates from the perceived inadequacy of the formal UME curriculum toward the Step 1 exam. This is partially because the environment cast by the Step 1 exam design promotes the memorization of commonly tested diseases and their associations [10]. Students are more open to engaging in formal activities of the clerkship curriculum because it typically aligns more closely with the Step 2 content [6], suggesting that reformatting of the curriculum and USMLE exams is necessary.

Third-party studying resources can cost up to hundreds of dollars for an annual subscription [15]. Despite the exorbitant costs, medical students at many schools are increasing their reliance on these commercial USMLE-oriented prep materials, increasing the prevalence of these products despite apprehension toward these examination-oriented learning goals [15]. Financial conflicts of interest are common, as private companies take advantage of the overemphasis on USMLE performance, exploiting students' anxieties about performing well and contributing significantly to medical student financial debt [10]. Besides the USMLE, the NBME itself sells a multitude of other assessment modalities commonly integrated into UME, such as subject exams, self-assessments, and the Customized Assessment Services (CAS) program, which allows medical schools to develop exams targeted toward individual curricula from a standardized question bank [16]. Students also report feeling overwhelmed by the number of resources and question whether the use of third-party materials will truly make them better physicians [13]. In one study evaluating factors associated with better licensing exam performance among Doctor of Osteopathic Medicine (DO) students, the use of commercial resources showed no improvement in USMLE or Comprehensive Osteopathic Medical Licensing Examination (COMLEX) scores [17]. Hence, promoting habits of regular and continuous learning, where students build upon their existing foundations of knowledge, is more beneficial to both student success and genuine professional growth [17].

Utilization of USMLE performance within the residency selection process

Overemphasis on USMLE performance reduces the significance of other areas that foster holistic clinical competence, such that medical students have less time to participate in research, servant leadership, direct patient care, volunteering, and community engagement [10]. For example, the dedicated period for studying for Step 1 and 2 exams reallocates time that could be used for clinically relevant activities. This reality is even more evident given that many students begin an "early dedicated period” by studying for the Step 1 exam while pre-clerkship courses are still in session [12], further distracting them from their medical school’s curriculum.

Additionally, given that higher USMLE exam performance is correlated with better residency match rates, increased emphasis has now been placed on Step 2 performance [8]. While the transition of Step 1 to pass/fail was meant to offer an opportunity to reduce stress and burnout, studies have demonstrated that the transition did not lead to a reduction of these issues in students preparing for the exam [9]. Hence, there is no reason to believe that this would bring a significant change if the emphasis simply shifted to another scored exam [8]. Transitioning to a pass/fail scoring schema alone is not enough; further reform is needed to realistically promote student well-being.

Increased focus on Step 2 and individual clerkship shelf exams has led to decreased involvement with the clerkship curriculum, as the anxiety over preparing for these assessments outweighs the interest in direct patient care [18]. Even within clerkship training, students’ abilities are often reduced to their performance on standardized exams rather than assessments that accurately reflect their competence and skills at a patient’s bedside [19]. This increased focus on the USMLE and NBME assessment system leads to compartmentalization of medical knowledge and skills rather than holistic integration that prepares students for graduate medical education (GME) [19]. 

Benefits of separation of USMLE from medical school curriculum

The USMLE exams were never intended to predict success during residency [20], which places their necessity for residency applications in doubt. Claims that argue for the usefulness of USMLE scores as screening tools due to their correlation with performance during residency training also lack consistency in the literature [21]. One systematic review revealed minimal correlation between Step 1 and Step 2 CK scores with other metrics used to rank students, including summative evaluations, professional assessments, and internship supervisor ratings across various specialties [22]. Another study examining the relationship between USMLE performance and ACGME milestones during the intern year of internal medicine residency found no correlation with Step 1 scores [21]. However, others have revealed that higher scores on all three Step exams may correspond to lower mortality rates and length of hospitalizations in the state of Pennsylvania [23]. In a similar vein, another study associated Step 2 CK scores with marginally improved sub-competency performance among emergency medicine residents [24]. Despite this, attributing patient outcomes to an individual physician's USMLE score represents an oversimplification of the intricate cooperative effort that is modern healthcare. Moreover, performance on a multiple-choice exam cannot be expected to thoroughly reflect a physician’s overall clinical competence; qualities like work ethic, time management, interpersonal skills, and even surgical precision are what truly separates a great test-taker from a superb physician. The concept of using milestone ratings and board certification exams as measures of competency still lacks definitive empirical support. There is no reliable gold standard for comprehensively gauging clinical practice, and further research is needed to show how these metrics truly correlate with quality patient care. As a result, the use of USMLE exams as a screening mechanism may be misguided and should cause alarm. Given that USMLE scores are loosely tied to actual residency performance and educational training outcomes, the validity of placing such high value upon them for residency consideration is called into question.

Because the USMLE exams are ultimately used for licensure, a transition to a pass/fail scoring system for all three exams would suitably fulfill their purpose; a numeric score is unnecessary for meeting their objectives [5]. Decreased emphasis on scores also broadens the range of career opportunities for medical students. Students previously at risk of scoring lower on USMLE exams would feel more confident in applying to competitive specialties [25], equalizing the playing field and promoting more holistic consideration of residency applicants [26].

Just as transitioning to curricula with pass/fail pre-clinical coursework has been associated with improved student well-being [27], such an outcome may also apply to licensing exams. With decreased stress surrounding USMLE scoring, there is hope for decreased reliance on parallel curricula, drawing away from a toxic Step 1 culture and allowing dedicated time for non-exam activities like research and extracurricular activities [20]. For example, to further circumvent the problems associated with Step 1, some institutions have already postponed the USMLE Step 1 exam after core clinical clerkships [28]. Furthermore, studies indicate that taking Step 1 post-clerkship is correlated with better performance, as experiences in clinical settings can provide valuable context that supplements the comprehension of basic science concepts [29], thereby promoting clinical reasoning through the correlation of foundational and clinical science topics [30]. In addition, students with professional clinical experience prior to matriculation tend to score higher on Step 1 and 2 exams, further supporting the notion that clinical context aids in integrating learned material into long-term memory [31]. Exposure to clinical practice fosters deeper understanding and better problem-solving skills, both essential for residency and future independent clinical practice [31]. Given that students often forget foundational science information after passing Step 1, our proposed system of postponing licensing exams may bolster retention as they progress through clinical years [28].

Reform of medical education is not a novel idea and in fact should be anticipated, as students and clinical practice itself evolve. In 2004, an initiative was launched to revise the USMLE's purpose and design to better align with the medical profession's values [16]. During this time, the Committee to Evaluate the USMLE Program (CEUP) developed recommendations for better integration of fundamental medical sciences within the clinical context for licensing exams to better emphasize clinical decision-making and to ensure that these assessments meet the evolving needs of medical education and licensure [32]. Furthermore, the permanent suspension of Step 2 Clinical Skills (CS) during the COVID-19 pandemic was partly due to the exam's costs and stress outweighing its benefits for residency evaluation [33]. Thus, from an organizational perspective, changes to the medical licensing system are not only possible, but to be expected. An increasing number of institutions are also encouraging students to take Step 1 post-clerkship; if postponing the exam until after clerkships is feasible, deferring it for an additional year is not such a radical proposition.

Navigating the separation of USMLE from the medical school curriculum

The transition to a separate licensing examination system, in which passing USMLE exams is not essential for medical school graduation and entry to residency, raises as many benefits as logistical considerations. Through our proposal, medical students would be expected to take both USMLE exams between March and June of their fourth year of medical school, following their Match Day decisions. The students will proceed to graduate from medical school based on their school performance and not the outcome of USMLE, which remains separate from examinations required by the program. This timeline of exam administration is similar to that used by other professional schools; for example, graduation from law school is not dependent on passing the General Bar Examination and instead prioritizes student competence during their legal training. While it may be argued that the stakes of the USMLE are higher than that of the Bar Examination, licensing exams assess only a limited scope of an aspiring physician’s capacity; they do not evaluate a medical student’s overall proficiency in providing holistic patient care, as this quality will be nurtured and refined during their residency training regardless of their USMLE score. Such a system for meeting graduation requirements is also demonstrated through pharmacy school programs and even within graduate medical education, as resident physicians are not required to pass their specialty-specific board exams to successfully complete their residency training [34].

**Figure 1:**
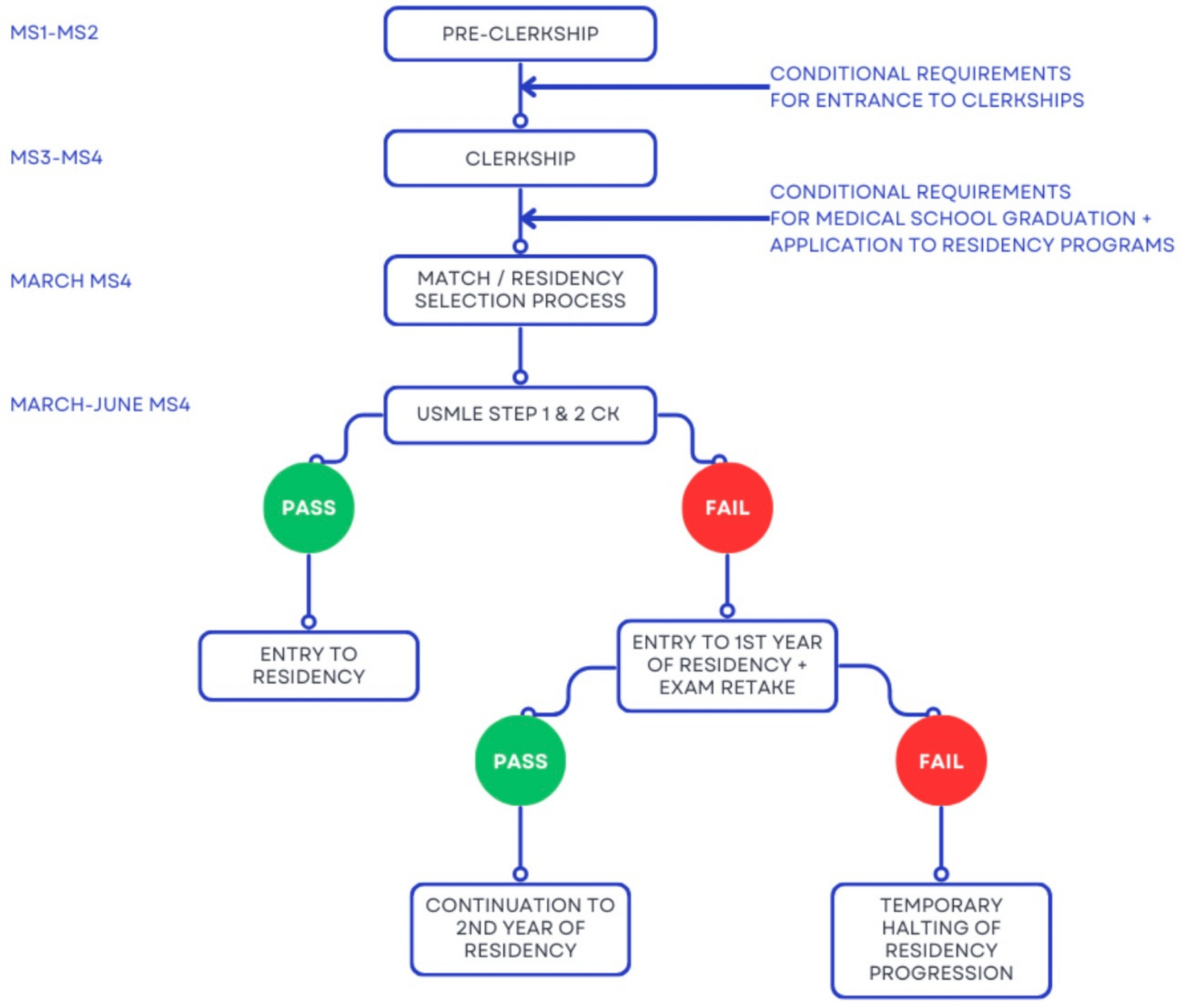
The proposed reformed timeline of USMLE exam administration MS: medical student. Image Credit: Authors' original creation.

Through our proposal, medical students may still be permitted to begin residency if they fail their exams on the first attempt, with the expectation that they will re-take their exams during their first year of residency. However, given the need for minimum qualification standards for remaining in clinical practice, such students would be unable to progress to the second year without a passing score. For these residents, a break from graduate medical education may be warranted, in which they may dedicate time toward studying for exams and potentially reapplying to an alternative program. To accommodate this and to allot ample time for success, the timeline for finishing all USMLE exams can be adjusted to a six-year span beginning at matriculation. A diagram outlining this proposed new timeline for licensing exams in relation to medical school education is shown in Figure 1.

One could consider the possibility that medical schools themselves could theoretically make the decision to detach USMLE completion from their graduation requirements, as the undergraduate medical curriculum is not determined by the NBME. However, an issue lies in the pressure to align with national licensure standards and the expectations of residency PDs. As a solution, medical schools could offer more flexibility beyond the “dedicated” exam preparation period. In response to the limitation of professional identity formation under current USMLE expectations, medical schools may also discuss with students at risk for failing Step 1 or 2 CK exams the option of pursuing alternate career paths in healthcare or related fields rather than applying to residency, while still being able to graduate with a medical degree. Though this raises the concern of diminishing the credibility and value of the degree itself, students are nevertheless afforded an opportunity to gain skills and contribute to the healthcare system outside of clinical practice, which is both a noble and necessary endeavor. This option would, of course, only be framed as a suggestion to students, and the priority of medical schools would be to ensure that the student achieves their professional goals, including those related to clinical practice. As a result, medical school advisors should offer additional academic support for students performing lower than expected on internal metrics in order to maximize their chances of passing both USMLE exams post-graduation.

Another important concern is maintaining student competence in preparation for residency. Studies suggest that performance on rigorous assessments administered throughout medical school can serve as predictors of Step 1 and Step 2 CK scores and significantly enhance the likelihood of success on these exams [35]. These assessments include customized comprehensive exams, such as those administered through the NBME CAS program, as well as subject exams, homegrown medical schools course examinations, and overall clerkship grades [2,36]. Additionally, students may be evaluated through their performance on clerkship oral exams and Objective Structured Clinical Examinations (OSCEs) [37], allowing for assessment of required patient care skills valued by residency programs. Some schools have implemented Transition to Residency Courses (TTR), in which students have the opportunity to develop essential skills in preparation for their ascension to GME [38]. These factors have long been used in predictive models to screen for students at risk of failing the USMLE exams [39,40]. In our proposed system, these assessments could be utilized by medical schools to ensure that students are making adequate academic progress to pass their licensing exams following graduation. Such a system also allows opportunities to reevaluate other criteria relevant to clinical competence within the context of medical school graduation requirements. For example, greater emphasis may be placed on the assessment of student professionalism through observation of interpersonal and communication skills [41,42]. These assessments are not intended to replace the need for standardized evaluation of medical student competence but rather would be utilized as internal metrics to ensure that students meet the necessary knowledge requirements and clinical proficiency to progress to their next level of training.

The virtue of this design is that it calls for reconsideration of the priorities used for residency selection in alignment with the overarching goals of both UME and GME [43]. The objective of this reformation is not to make medical school simpler [44] but to cultivate a conducive learning environment where students can be fully immersed and engaged with the foundations of medicine and their school curriculum rather than worried about the USMLE examination from day one. Refinement of the curriculum and integration of foundational and clinical sciences is not only essential but also aligns with the original objectives of medical education and licensure exams.

Other merits of this reform include boosting student well-being and allowing full immersion in medical school without the distractions of a parallel curriculum generated by the burden of Steps examinations and its relevance to residency selection. A new curricular system allows educators to further explore topics that resonate with medical practice and the healthcare system, even if not considered “high-yield” subjects, such as population health and health policy, medical ethics, health disparities, social determinants of health, and bedside clinical skills [8,43]. These topics should be taught within the context of patient-centered care and the physician’s obligations to society [45].

A key feature of this approach to medical education is the spiral integration of foundational and clinical science content, featuring consistent revisiting of material throughout the curriculum across disciplines and time [46]. Such repetition and integration would facilitate the retention of material; drawing connections between new and previously covered concepts provides a framework for breaking down the barrier between pre-clerkship and clerkship education [30].

Improved integration will also help students navigate away from the “binge and purge” approach to basic science topics by emphasizing the interrelatedness of all aspects of medical education [1]. Additionally, involvement in clinical practices during the first years of medical school and revisiting foundational science material in later years will allow for deeper comprehension [31] and hence better preparedness for licensing exams required during post-graduation.

Another merit of our proposal is the renewed ability of students to allocate more time and effort to engage in scholarship and service learning that strengthen medical reasoning ability and allow them to explore their professional identities as future physicians [47]. While research experience may have little predictive power as to a given medical student’s success during residency [48], there are nevertheless many benefits to engaging in research while in medical school, provided that students have the time and support to genuinely explore their interests. Exposure to research during UME not only encourages the development of critical thinking and passion for lifelong learning but also introduces students to the significance and application of evidence-based medicine [49]. Similarly, offering students the time to grow skills for leadership positions would help them expand team-building skills and increase authentic engagement in passion projects and volunteer experiences, which directly benefit local communities. By reducing the influence of parallel curriculum created with external motivators for USMLE scores on residency match success, students can better indulge in intrinsic motivations for becoming compassionate humanistic physicians and find greater fulfillment in their medical education, career choice, and professional development. A curriculum that encourages intrinsic motivation, curiosity, and professional identity formation will better equip students to navigate the ambiguity and uncertainties of the medical field [42,46]. It is unrealistic to expect students to engage meaningfully with the school curriculum and not parallel curriculum aligned with the contemporary overreliance on commercial board-prep resources [50]. Therefore, the separation of USMLE exams from the medical school curriculum allows for the exploration of topics that are equally relevant to comprehensive physician competence, which may be suppressed in the current USMLE-focused parallel curriculum and education system, which prioritizes exam performance above all other competencies.

Unintended consequences of disconnecting USMLE exams from MD curriculum

The most desirable outcomes of separating USMLE exams from the MD curriculum are mastery and retention of the foundational sciences as well as optimal integration with experiential clinical learning, reducing the need for a parallel curriculum for performing well in USMLE exams. Several arguments are resisting our proposed change to USMLE scheduling, one of which relates to student preparedness as they approach clinical rotations. Studying for Step 1 can consolidate medical knowledge, effectively instilling confidence prior to clerkships. In addition, pass rates for Step 1 have declined since the transition to a pass/fail grading system, dropping from a 97% pass rate in 2020 to 90% in 2023 [51]. However, since fluctuations in score performance over the years are common [4], it may be premature to determine if this decline is indicative of a broader trend of declining importance of foundational science for clinical practice. Students’ concerns and uncertainty in this regard can be alleviated with more consistent and deliberate integration of basic sciences and clinical knowledge by curriculum developers, allowing for optimal connection of medical knowledge with clinical practice. In addition, the noted decline in the Step 1 pass rate following the switch to pass/fail may reflect two features: a complacency that students felt in regard to their knowledge and a disconnect between test content and didactic curricula. While pre-clerkship medical curricula begin to increasingly cover material on clinical management, students become increasingly frustrated by the continued emphasis of Step 1 on foundational science topics, which may be deemed as less clinically relevant. This discrepancy highlights a desperate need to reevaluate the topics tested on the examination to better align and reflect the didactic curriculum of schools.

Another caveat is found in the potential lack of identification of students who may be at risk of failing the USMLE exams, making them less likely to receive early academic support [52]. Strategies to account for this may involve the implementation of academic coaching to ensure appropriate academic progress throughout medical school education. There may also be concern on the part of state boards that postponing USMLE exams would cause difficulty in assessing whether a candidate would meet the criteria for licensure as a qualified physician. However, our proposed system does not seek to undermine these standards for competent clinical practice. First-year medical trainees would still be required to pass licensing exams in order to be able to independently practice within a given state; these exams would simply be undertaken at a slightly later point in their career trajectory. Additionally, there are those who doubt that the transition to the pass/fail system has genuinely improved student well-being. Some studies suggest that there has been no significant reduction in stress levels since Step 1 became pass/fail [20,53]. However, the similarity in stress levels could be attributed to the resultant shift toward increased emphasis on Step 2 or research activities [20].

Perhaps the most significant argument against the exclusion of USMLE performance from residency selection criteria is that PDs may resort to other “standardized” metrics for screening applicants, potentially promoting inequities in the residency selection process [54,55]. These metrics could include membership in Alpha Omega Alpha (AOA) or Gold Humanism Honor Society (GHHS), clerkship grades, medical school ranking, research publications, and personal knowledge of the applicant through participation in audition rotations [56,57]. The reliance of residency programs on using alternate metrics may increase competition for resources within cohorts, effectively altering interpersonal dynamics and contributing to stress as access to research projects and leadership positions becomes limited [57]. To address these issues, residency PDs should be more transparent about the criteria they value in applicants for their programs and specialties [18], and medical schools should provide greater support for students engaging in activities to enhance the well-roundedness of their applications [44]. Although relying on an assortment of curricular and extracurricular experiences can increase diversity in the residency cohort, it also opens the door for over-reliance on personal connections and nepotism, undermining the justness of the candidacy. There is also concern that increased reliance on internal metrics reported by medical schools may lead to grade inflation to increase student likelihood of a successful residency match. However, through our proposed system, such internal metrics should still be related to objective measures, such as comprehensive assessments during the pre-clerkship curriculum and adequate performance on shelf exams during clerkship training. These measures should not be weighted so heavily as to replace USMLE scores and cause undue stress on medical students but instead included as one of many holistic measures evaluated by medical school administrators and residency PDs to gauge student preparedness for graduate medical education.

Furthermore, the supposedly objective factors and metrics used by PDs do not have high predictive value for performance during residency [21], making a more holistic approach an attractive solution. There is additional concern regarding the decreased diversity of residency programs due to disadvantages faced by socially and economically disadvantaged students, DO students, and international medical graduates (IMGs). This can partially be remedied with adjustments to the COMLEX examination schedule that emulate the proposed changes to USMLE exams, thereby offering DO students more flexibility and autonomy to pursue supplemental activities to bring a more diverse array of experiences similar to MD schools for residency applications. Additionally, it is possible that the lack of “objective” metrics may yield increased discrimination and dependence on stereotypes when considering disadvantaged medical students for residency positions [58].

Moreover, USMLE scores previously created a standardized metric for IMGs and DO students, giving them an opportunity to demonstrate their capabilities alongside MD applicants [18,59]. Without this criterion, IMGs may be placed at a disadvantage as factors like medical school rank and MSPE letters are not applicable, and securing Visas for elective rotations is often challenging [59]. A viable solution would be for medical schools and residency PDs to provide greater transparency regarding student strengths and overall criteria for residency selection, as mentioned previously. Furthermore, new legislation introduced as a solution to the growing shortage of physicians allows IMGs to bypass US residency training in certain states; this accommodation is dependent on their obtaining Educational Commission for Foreign Medical Graduates (ECFMG) certification as well as at least three years of experience in medical practice [60]. Over time, ECFMG certification criteria for IMG applicants may require further modification as well.

Conclusion 

The current system of integrating USMLE exams so intricately within medical school curricula has led to a USMLE-oriented parallel curriculum, unnecessary amounts of stress, burnout, and overall detriment toward students’ professional development and well-being. Given the original intention of such licensing exams to serve as threshold markers for medical students’ readiness for clinical practice, it is not necessary for them to serve as prerequisites for graduation. Medical school graduates should be eligible for supervised clinical practice, regardless of USMLE completion, as exemplified by other professional schools. As reform of licensing exam implementation continues, it may be worthwhile to consolidate Step 1 and 2 CK into one decisive exam to determine eligibility to enter supervised practice, while retaining Step 3 during residency training and specialty board certification to assess readiness for unsupervised clinical care. We urge the USMLE, NBME, FSMB, ACGME, AACOM, and ECFMG to consider reassessment of the timing of USMLE exams in relation to medical school graduation. Through our proposal for postponement of licensing exams, we aim to promote an approach to medical school curricular reform that emphasizes a return to the original tenets of the medical profession, allowing for increased focus on professional identity formation as well as consistency in the integration of foundational and clinical sciences throughout UME.
